# Mediterranean blue tits as a case study of local adaptation

**DOI:** 10.1111/eva.12282

**Published:** 2015-10-27

**Authors:** Anne Charmantier, Claire Doutrelant, Gabrielle Dubuc‐Messier, Amélie Fargevieille, Marta Szulkin

**Affiliations:** ^1^ Centre d'Ecologie Fonctionnelle et EvolutiveCampus CNRSMontpellierFrance; ^2^ Département des sciences biologiquesUniversité du Québec à MontréalSuccursalle centre‐villeQCCanada

**Keywords:** adaptive divergence, *Cyanistes caeruleus*, environment heterogeneity, genomic differentiation, genotype–environment association, heritability, isolation‐by‐environment, phenotypic differentiation, phenotypic plasticity

## Abstract

While the study of the origins of biological diversity across species has provided numerous examples of adaptive divergence, the realization that it can occur at microgeographic scales despite gene flow is recent, and scarcely illustrated. We review here evidence suggesting that the striking phenotypic differentiation in ecologically relevant traits exhibited by blue tits *Cyanistes caeruleus* in their southern range‐edge putatively reflects adaptation to the heterogeneity of the Mediterranean habitats. We first summarize the phenotypic divergence for a series of life history, morphological, behavioural, acoustic and colour ornament traits in blue tit populations of evergreen and deciduous forests. For each divergent trait, we review the evidence obtained from common garden experiments regarding a possible genetic origin of the observed phenotypic differentiation as well as evidence for heterogeneous selection. Second, we argue that most phenotypically differentiated traits display heritable variation, a fundamental requirement for evolution to occur. Third, we discuss nonrandom dispersal, selective barriers and assortative mating as processes that could reinforce local adaptation. Finally, we show how population genomics supports isolation – by – environment across landscapes. Overall, the combination of approaches converges to the conclusion that the strong phenotypic differentiation observed in Mediterranean blue tits is a fascinating case of local adaptation.

## Introduction

Evolutionary biologists are primarily interested in understanding the processes that explain the origin and maintenance of biological diversity. Phenotypic differences within a given species can have at least three main origins: genetic drift, phenotypic plasticity or evolutionary divergence, also called adaptive divergence. For populations to adapt to their local environment, that is for local adaptation to occur, divergent selection between habitat patches should result in higher relative fitness of resident versus immigrant genotypes (Williams 1966). This process of divergent adaptation, although primarily triggered by opposing forces of natural selection, also involves a complex interplay between selection, gene flow, genetic drift and the presence of genetic variation in fitness traits (Hedrick 2000; Savolainen et al. 2013). Hence, demonstrating an adaptive process requires several steps that involve knowledge on the phenotypic variation, the populations' genetic properties and dynamics, the quantitative genetic (co)variation of adaptive traits and the characterization of the shape, direction and strength of natural (social and sexual) selection acting on these traits.

The magnitude of adaptive divergence is theoretically expected to increase with the amount of genetic variation within populations (Fisher 1930; Lande 1980) and the level of environmental divergence between populations (Endler 1977). Adaptive divergence is also expected to be greater when genetic drift (Lande 1976; Hereford 2009) and gene flow between populations (Slatkin 1985; Lenormand 2002) are lower. However, the interplay between ecological and evolutionary processes can substantially complicate this general picture. For instance, although gene flow is generally predicted to have a homogenizing effect counteracting phenotypic diversification (Slatkin 1987; Garcia‐Ramos and Kirkpatrick 1997; Lenormand 2002), gene flow can reversely promote adaptive divergence by counteracting inbreeding depression or by increasing genetic variation, and thus adaptive potential (Garant et al. 2007). As a consequence of this complexity in the interactive processes, the study of local adaptation is ideally multifaceted and multidisciplinary (Kawecki and Ebert 2004), a challenge which is more realistically faced in experimental systems than in free‐living populations. One of the best ways to investigate higher performance of resident versus immigrant organisms in a given habitat is to use reciprocal transplants or common garden experiments because these approaches allow maximizing the power to reveal genotype‐by‐environment interactions. Such experiments have provided so far the most extensive contributions to our understanding of processes involved in adaptive divergence (see Blanquart et al. 2013 for a review). However, they are not easily applicable in many organisms and do not always allow an accurate evaluation of the relative importance of selection versus gene flow or genetic drift, in particular in highly mobile organisms occupying a heterogeneous environment (see e.g. Merilä and Hendry 2014 for a review in the context of a response to climate change).

The study of the origins of biological diversity between species has provided several emblematic examples of adaptive divergence (Schluter 2000). Two prominent examples are the adaptive radiation in beak size and shape in the Galapagos Darwin's finches (e.g. Grant 1986) and the explosive diversification of cichlid fishes in the lakes of East Africa (e.g. Kocher 2004). Overall, studies of species complexes have provided sufficient examples to conclude that adaptive radiation is widespread (Schluter 2000; Gavrilets and Losos 2009). However, when it comes to understanding phenotypic variation at smaller temporal and spatial scales, there is much less compelling examples, in part because the above‐mentioned complexity of processes makes it very difficult to demonstrate that phenotypic divergence is adaptive and that it has a genetic basis.

Although historically, geographic isolation of populations was seen as a prerequisite for their adaptive divergence (Mayr 1963), it has now long been recognized that reproductive isolation can evolve even between populations connected by gene flow whenever divergent selection is strong relative to gene flow (Maynard Smith 1966; Rice and Hostert 1993). However, the realization that such divergence in spite of gene flow can occur at microgeographic scales, that is within the range of the organism's dispersal distance, is only just arising (Postma and Van Noordwijk 2005; Milá et al. 2010; Richardson et al. 2014). This is surprising given that some classical examples of fine scale adaptation, such as the adaptive variation in colour morphs of the Peppered Moth *Biston betularia,* date back several decades (Saccheri et al. 2008).

We aim here at showing how the Mediterranean blue tit *Cyanistes caeruleus* study system is an equally fascinating example of phenotypic divergence. We will outline the story of a long‐term project where we tested for phenotypic and genetic divergence as well as for possible local adaptation of bird populations using a diversity of complementary approaches – from the study of phenotypic differentiation to the recent development of ecological genomics. This project started with the erection of nest boxes by Jacques Blondel in 1975 in Mediterranean oak forests of Provence (Southern France) and on the island of Corsica, with the purpose of comparing breeding strategies between mainland and island populations (Blondel 1985). The long‐term monitoring of blue tits in these forests led to the striking observation that populations as close as 24 km differed in their breeding phenology by up to 1 month, depending on the type of vegetation they use for reproduction (Blondel et al. 1999). Although the habitat‐specific phenotypic differences were soon found salient (Blondel et al. 1993; Lambrechts et al. 1996, 1997a), understanding the processes that lead to this intraspecific biodiversity is still an ongoing objective. Indeed, if one is to avoid the Panglossian paradigm whereby adaptation is considered a fundamental assumption (Gould and Lewontin 1979), one needs to consider alternative (and nonexclusive) adaptive (including plasticity) and nonadaptive (including drift) processes when testing for an adaptive divergence. Moreover, the integration of phenotypic, genetic and fitness data is required to confidently confirm evolutionary adaptation at the genetic level (Barrett and Hoekstra 2011) and is at the core of current research in this study system. Within the abundant literature published from this study system, we will focus here on the main findings that provide tests for an adaptive origin to the strong phenotypic differentiation observed across heterogeneous Mediterranean landscapes.

After briefly presenting the model system, we first summarize the main phenotypic divergence observed in blue tit populations living in forests dominated either by deciduous or evergreen trees. For each divergent trait, we review the evidence obtained from aviary/common garden experiments regarding a possible genetic origin to the observed phenotypic differentiation as well as evidence for heterogeneous selection. Second, we argue that most phenotypically differentiated traits display heritable variation, a fundamental requirement for evolution to occur. Third, we review evidence for nonrandom dispersal, selective barriers and assortative mating as processes that could reinforce local adaptation. Fourth and finally, we show how population genetics and ecological genomics support isolation – by – environment (IBE) across habitats. As this study is published in a Special Issue that aims at highlighting women's contribution to basic and applied evolutionary biology, we have added some personal comments on women's position in this long‐term project (Box 1).

Box 1 **Personal reflections**

Research presented here is based on the Mediterranean blue tit study system that was started forty years ago by J. Blondel. The project was originally developed by men only, in particular by J. Blondel, P. Perret and M. Lambrechts. In the past 10 years, the sex ratio of the team has largely become equal. We do not feel that this change in sex ratio led to any more changes in research questions than expected due to the inclusion of any new researcher in a team. However, more equal sex ratios may have resulted in more diverse ways of working with students and positively impacted group team working in the field and in consequent research work. We do not intend to draw general conclusions based on the progression of this single scientific group, yet we wish here to share some personal feelings as women working in the field of evolutionary biology.

Working in a country (France) where 80% of women claim to be victims of sexism at their workplace (2013 poll on 15 000 women by the Higher Council of Professional Equality (CSEP)), we feel privileged to state that the authors of this article have not experienced overt gender‐based discrimination in their PhD, postdoctoral or senior research years. However, this does not exclude the occurrence of more subtle or hidden preconceived biases about the professional capacity of males and females that are still common worldwide. These are often reflected in recruitment outcomes when applying for permanent/tenure track positions (Reuben et al. 2014). In addition, the fact that tenured positions tend to be secured at an increasingly older age, often at a time when fertility curves in women are declining, only aggravates women's underrepresentation in science (Ceci and Williams 2011). While in‐depth studies quantifying gender bias in science and identifying its causes and consequences are available elsewhere (Ceci and Williams 2011; Williams and Ceci 2012; Reuben et al. 2014; Leslie et al. 2015), here we highlight how, as women, we interacted with the requirements of scientific work involved in this long‐term research project, and how we believe to have created a positive working environment from which the research programme, women and men researchers, and their children, have all benefitted from.

Conducting long‐term research in ecology implies being committed to fieldwork for several weeks (if not months) away from home annually. As several female researchers reported beforehand (e.g. Mcguire et al. 2012), we reconciliate the two by occasionally bringing our children to the field and by relying on our partners and family for engaging in joint child rearing. This is true for both mothers and fathers working in our research group. At the same time, flexibility is an equally important aspect of running a large‐scale field project with a larger number of fieldworkers. Thus, parental leaves of team members were never an issue as field logistics were always prepared well ahead of time and contingency plans were available. Importantly, special efforts were made to accommodate researchers with small dependents (*i.e*. their children), thereby substantially improving the suitability of the work environment for mother and father ecologists (for an excellent analysis of the challenges faced by women ecologists, see the study of Mcguire et al. 2012). These steps not only incredibly benefitted each individual researcher at such special point in their life (Williams and Ceci 2012 and references therein), but it also strengthened the team of fieldworkers available each year, and allowed to foster a sense of continuity, inclusion and identity in promoting the long‐term dimension of the programme.

**Figure B1:**
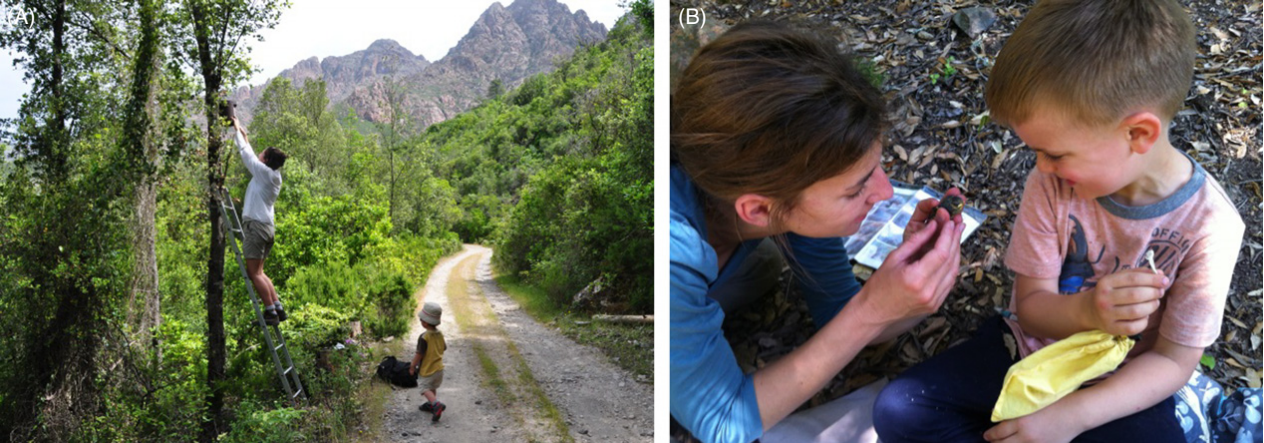
Bringing children to the field. (A) The daily nest box monitoring in the Fango valley and in good company. (B) Allowing children to participate in fieldwork increases their awareness of the natural environment, which is often a stepping stone to set up science outreach projects. Photo credit: (A) F. Laurière; (B) M.‐O. Beausoleil.

In terms of fieldwork practicalities, the only noticeable change following this sex‐ratio transition was a lowering of the highest nest boxes to allow access to all, which made life on the Blue tit field easier for both men and women. With more PI women in the field, fieldwork became a more family friendly adventure (Fig. B1(A)), allowing mothers – and fathers – to find a new middle ground that focuses on fieldwork. From the child's perspective, coming to the field is an incredibly enriching experience (Fig. B1(B)), as they are exposed to wilderness we are increasingly separated from in our daily lives. Equally importantly, they can also witness their parents in their professional role, in a unique environment and in a setting void of gender roles. Including children into our fieldwork routines often increased our participation in science outreach projects in kindergartens and schools while back at home, but also within the field site communities. Although quantitative data are needed to support this claim, we believe that transgressing traditional divides of work and life by welcoming children in the field sensitizes us to further participate in educational science outreach projects and thus directly impacts our communities. We hope this testimony will provide encouragement to young women and men and convince them that embarking in a field project is rewarding both professionally and personally and that it can be made compatible with a fulfilling family life.

## A model species in a model environment

The geographic configuration of Mediterranean landscapes, characterized by a heterogeneous, fine‐grained mosaic of habitats, provides an exceptional study system for investigating plastic and adaptive responses in spatially structured populations (Blondel and Aronson 1999; Blondel et al. 2010). In this system, we have focused on the variability expressed by a passerine bird, the Blue tit, whose ecological niche is preferentially linked to oak woodlands. The Blue tit is present in a narrower range of habitats compared to the Great tit *Parus major* (Snow 1954; Lack 1971), and yet is a common breeder throughout Europe and western Asia where it readily breeds in nest boxes, which makes it an ideal model to study adaptation to environmental heterogeneity. In our study area, the breeding activity of about 350 blue tit pairs is monitored each year in more than 1000 nest boxes erected across eight study sites, in a series of habitats that strongly differ in being dominated by patches of either deciduous (*Quercus pubescens*) or evergreen (*Quercus ilex*) oaks (Box 2). In deciduous forest patches, the leafing process occurs ca. 1 month (3–5 weeks depending on the scale of analysis) earlier compared to evergreen forests, and all leaves are renewed. This results in an earlier and more abundant caterpillar peak in deciduous patches (Zandt et al. 1990; Dias et al. 1994), which are considered as higher quality breeding habitats (Blondel et al. 2006). These differences between habitats are clearly visible to the human eye (Box 2) and can be mapped at a large scale using vegetation reflectance data from satellite sensors, the latter correlating remarkably well with the number of either type of oak quantified on the ground within a 50 m radius from each nest box (Szulkin *et al* 2015). Apart from the notable heterogeneity of the Mediterranean landscape, these study populations are also situated at the species range‐edge, a position known to be conducive to geographic differentiation (Mayr 1970; Endler 1977). Hence, the study of these populations may be of particular conservation value because they represent significant components of intraspecific biodiversity (Hardie and Hutchings 2010) and potential sources of evolutionary innovation and persistence during rapid environmental change such as global warming (Sexton et al. 2009).

Box 2

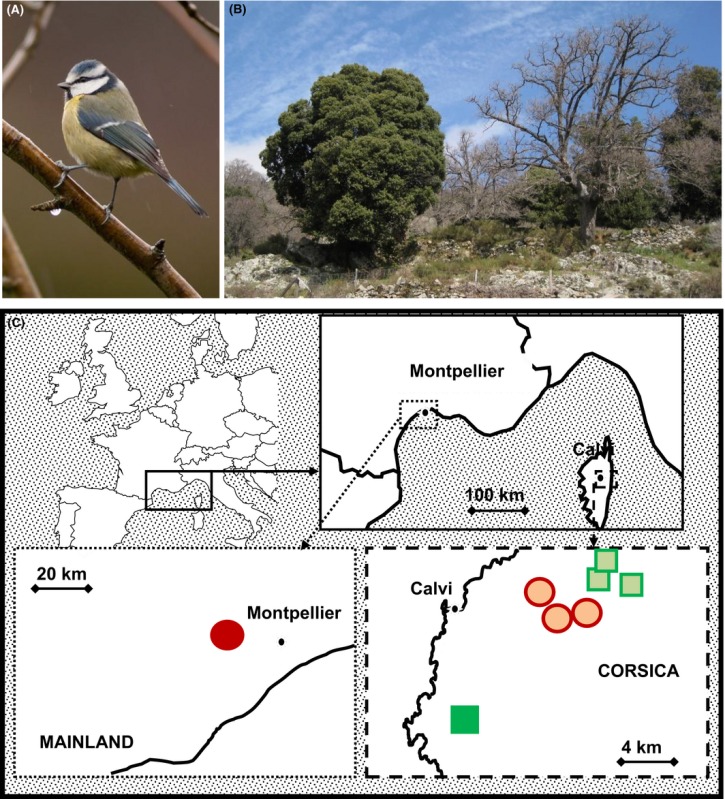

(A) The Blue tit *Cyanistes caeruleus* occupies habitats dominated by (B) the evergreen holm oak *Quercus ilex* or the deciduous downy oak *Quercus pubescens* (deprived of leaves in winter). (C) Locations of the study sites of blue tit populations monitored on the French mainland and in Corsica. Circles denote deciduous sites (*Quercus pubescens*) and squares evergreen sites (*Quercus ilex*). Phenotypic and pedigree data have been collected since 1979 in E‐Pirio (Lat: 42.38; Long: 8.75 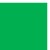), 1991 in D‐Rouvière (43.66; 3.67 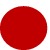), 1993 in D‐Muro (42.55; 8.92, three sites of Avapessa, Feliceto, Muro 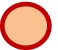) and 1998 in E‐Muro (42.59; 8.96, three sites of Arinelle, Filagna and Grassa, 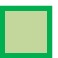). Photo credit: (A) S. Caro; (B) A. Charmantier.

Over the last forty years, the Montpellier blue tit group has ringed 6653 blue tit parents and 33 489 blue tit chicks in these study populations. This sustained hard work aimed at elucidating the phenotypic variation displayed by blue tits at three spatial scales (Blondel et al. 2006): mainland versus Corsica (min. distance of 85 km off mainland Italy and 170 km off the French mainland), a scale that is not the main focus of the present study (but more details about this inference level can be found in Dias and Blondel 1996; Lambrechts et al. 1997b; Blondel et al. 2001; Doutrelant et al. 2001); Muro versus Pirio valleys (24.1 km between D‐Muro and E‐Pirio, where ‘D’ stands for Deciduous and ‘E’ for Evergreen), and three deciduous plots (D‐Muro) versus three evergreen plots (E‐Muro) within the Muro valley (separated by 5.6 km). Although these distinct study plots within the Muro valley have been detailed in some previous publications (e.g. Lambrechts et al. 2004), we have pooled their blue tit populations here as they never display any significant differentiation, neither phenotypically nor genetically.

Because of the low rate of capture–recapture data using multisites, our knowledge on natal and breeding dispersal in the Blue tit remains approximate, with very different results in different studies. For example, while a maximum dispersal distance of 14.5 km was recorded in Belgium (Tufto et al. 2005), an earlier study over 1200 km² in Germany revealed that natal dispersal could reach distances of 24 km in males and 470 km in females (Winkel and Frantzen 1991). Note that in the same study, breeding dispersal (that is the distance between successive reproductive events) reached maximal distances of 0.75 km for males and 37 km for females. At the same time, the mode of the distribution is likely to be much smaller, especially in Corsican blue tits (Blondel et al. 1999), and most of our dispersal events are recorded within sites even when other populations are monitored close by. Over the last 17 years of ringing in Corsica, we observed 2 dispersal events between D‐Muro and E‐Muro (5.6 km), 2 reverse events between the same sites, and none between the Muro valley and E‐Pirio (24.1 km).

## Phenotypic divergence across blue tit populations

The phenotypic divergence measured across the four main study populations is detailed for life history, morphological, behavioural, vocal and colour traits in Table 1. Considering the massive amount of information regarding phenotypic differences, we only briefly discuss below the most remarkable results in the context of possible adaptive divergence. We also review attempts to test for the plastic/genetic origin in the described trait divergences as well as evidence for heterogeneous selection.

**Table 1:** Phenotypic divergence between blue tit populations in deciduous (D‐) and evergreen (E‐) patches on the mainland (D‐Rouvière) and in Corsica (D‐Muro, E‐Muro, E‐Pirio). Mean, sample size (*n,* number of individuals, except for survival probability where it is number of years), variance and coefficient of variance (CV) are provided for survival probability, four reproductive traits measured on first broods, four morphological traits measured on breeding individuals, four song traits recorded on breeding birds during the egg laying period, two behavioural traits measured just before or during the breeding period, and five colour traits measured on breeding birds during the whole reproductive period. For laying date, 1* *= 1st march. For number of fledglings, all broods included in an invasive experiment (e.g. cross‐fostering, increased cost) were removed. For tarsus length, only the twelve best measurers (of 70 in total, minima of 200 measures and a 90% repeatability) were retained. For life history and morphological traits, data were collected between the first year of monitoring, and 2014, except for annotated estimates driven from the literature. Data collection spanned 1998–2001 for song traits, 2011–2014 for personality traits and 2005–2013 for colour traits

First year of monitoring		D‐Rouvière	D‐Muro	E‐Muro	E‐Pirio
		1991	1993	1998	1976
Life history traits					
Adult survival probability*	Mean (*n*)	0.511 (8)	0.391 (6)		0.574 (14)
	Variance	0.005	0.016		0.005
Laying date	Mean (*n*)	39.12 (1773)	38.56 (1233)	48.21 (640)	70.08 (1920)
	Variance	58.89	65.36	57.39	52.70
Clutch size	Mean (*n*)	9.95 (1769)	8.50 (1235)	7.12 (638)	6.61 (1913)
	Variance (CV)	3.53 (0.19)	2.97 (0.20)	1.65 (0.18)	1.53 (0.19)
Incubation period (days)	Mean (*n*)	14.70 (1433)	13.62 (1161)	13.06 (587)	13.87 (1798)
	Variance (CV)	9.90 (0.21)	4.80 (0.16)	4.42 (0.16)	4.39 (0.15)
Number of fledglings	Mean (*n*)	6.24 (1445)	6.60 (1092)	4.14 (557)	4.15 (1273)
	Variance (CV)	16.48 (0.65)	8.67 (0.45)	9.15 (0.73)	6.56 (0.62)
Morphological traits					
Male Body mass (g)	Mean (*n*)	11.01 (1465)	9.82 (1032)	9.66 (455)	9.37 (1607)
	Variance (CV)	0.28 (0.05)	0.23 (0.05)	0.19 (0.04)	0.21 (0.05)
Female Body mass (g)	Mean (*n*)	11.01 (1713)	9.66 (1153)	9.47 (480)	9.23 (1616)
	Variance (CV)	0.57 (0.07)	0.28 (0.05)	0.22 (0.05)	0.31 (0.06)
Male Tarsus length (mm)	Mean (*n*)	17.00 (1227)	16.52 (578)	16.42 (198)	16.27 (789)
	Variance (CV)	0.17 (0.02)	0.23 (0.03)	0.18 (0.03)	0.20 (0.03)
Female Tarsus length (mm)	Mean (*n*)	16.44 (1432)	16.05 (614)	15.99 (224)	15.84 (798)
	Variance (CV)	0.18 (0.02)	0.18 (0.03)	0.25 (0.03)	0.18 (0.03)
Male Wing length (mm)	Mean (*n*)	67.21 (1418)	63.26 (1033)	63.32 (443)	63.61 (1527)
	Variance (CV)	3.24 (0.03)	3.08 (0.03)	3.00 (0.03)	2.46 (0.02)
Female Wing length (mm)	Mean (*n*)	64.44 (1647)	60.81 (1138)	60.83 (471)	60.70 (1503)
	Variance (CV)	2.83 (0.03)	2.55 (0.03)	2.52 (0.03)	1.85 (0.02)
Male Beak‐nostril length (mm)	Mean (*n*)	6.54 (1310)	6.55 (965)	6.66 (415)	6.56 (1217)
	Variance (CV)	0.13 (0.05)	0.16 (0.06)	0.14 (0.06)	0.16 (0.06)
Female Beak‐nostril length (mm)	Mean (*n*)	6.70 (1518)	6.82 (1060)	6.83 (446)	6.72 (1176)
	Variance (CV)	0.14 (0.06)	0.19 (0.06)	0.14 (0.05)	0.16 (0.06)
Behavioural traits					
Average male repertoire size reported in one morning†	Mean (*n*)	3.5 (14)	4.1 (20)		4.7 (12)
	Variance (CV)	1.25 (0.32)	1.46 (0.29)		3.84 (0.42)
Maximal song frequency‡ (Hz)	Mean (*n*)	7788 (93)	8339 (168)		8138 (133)
	Variance (CV)	29 2681 (0.07)	45 1584 (0.08)		33 5241 (0.07)
Song duration‡ (s)	Mean (*n*)	1.93 (93)	1.57 (168)		1.33 (133)
	Variance (CV)	0.58 (0.39)	0.69 (0.53)		0.38 (0.46)
Silence duration‡ (s)	Mean (*n*)	0.06 (93)	0.07 (168)		0.09 (133)
	Variance (CV)	0.0004 (0.33)	0.0004 (0.29)		0.0004 (0.22)
Male Handling aggression score (0–3)	Mean (*n*)	1.54 (81)	1.82 (339)	1.70 (223)	1.68 (282)
	Variance (CV)	0.84 (0.59)	0.83 (0.50)	0.85 (0.54)	0.96 (0.58)
Female Handling aggression score (0–3)	Mean (*n*)	0.96 (88)	1.58 (376)	1.25 (227)	1.31 (303)
	Variance (CV)	0.77 (0.92)	0.96 (0.62)	0.89 (0.75)	0.95 (0.74)
Male openfield speed (cm/s)	Mean (*n*)		15.00 (81)	13.14 (67)	11.69 (65)
	Variance (CV)		61.12 (0.52)	58.70 (0.58)	70.21 (0.72)
Female openfield speed (cm/s)	Mean (*n*)		13.09 (105)	11.11 (66)	9.84 (82)
	Variance (CV)		75.14 (0.66)	52.82 (0.65)	30.64 (0.56)
Colour ornament traits					
Male Blue Brightness	Mean (*n*)	16.6 (886)	15.4 (472)	16.1 (297)	15.4 (498)
	Variance (CV)	26.5 (0.31)	20.2 (0.29)	23.2 (0.30)	20.0 (0.30)
Female Blue Brightness	Mean (*n*)	14.1 (949)	13.13 (515)	13.4 (305)	12.4 (519)
	Variance (CV)	28.9 (0.38)	15.0 (0.30)	15.2 (0.29)	16.6 (0.33)
Male UV–Blue Hue	Mean (*n*)	376.3 (886)	370.1 (472)	377.0 (297)	377.6 (498)
	Variance (CV)	129.0 (0.03)	93.1 (0.03)	179.5 (0.04)	149.4 (0.03)
Female UV–Blue Hue (nm)	Mean (*n*)	387.9 (949)	381.2 (515)	384.3 (305)	384.2 (519)
	Variance (CV)	131.9 (0.03)	203.4 (0.04)	266.4 (0.04)	152.0 (0.03)
Male Blue UV Chroma (nm)	Mean (*n*)	0.38 (886)	0.39 (472)	0.37 (297)	0.38 (498)
	Variance (CV)	0.0013 (0.09)	0.0014 (0.10)	0.0007 (0.073)	0.0013 (0.10)
Female Blue UV Chroma	Mean (*n*)	0.34 (949)	0.35 (515)	0.34 (305)	0.34 (519)
	Variance (CV)	0.0011 (0.10)	0.0013 (0.10)	0.0009 (0.086)	0.0011 (0.10)
Male Yellow Brightness	Mean (*n*)	17.0 (854)	15.7 (472)	16.1 (297)	16.4 (500)
	Variance (CV)	12.0 (0.20)	11.3 (0.21)	12.5 (0.22)	11.1 (0.20)
Female Yellow Brightness	Mean (*n*)	17.1 (912)	16.5 (523)	16.7 (310)	17.0 (528)
	Variance (CV)	15.1 (0.23)	12.6 (0.22)	9.9 (0.19)	12.7 (0.21)
Male Yellow Chroma	Mean (*n*)	0.62 (854)	0.82 (472)	0.69 (297)	0.81 (500)
	Variance (CV)	0.03 (0.27)	0.03 (0.20)	0.02 (0.23)	0.02 (0.18)
Female Yellow Chroma	Mean (*n*)	0.61 (912)	0.74 (523)	0.65 (310)	0.70 (528)
	Variance (CV)	0.03 (0.28)	0.02 (0.20)	0.03 (0.26)	0.02 (0.18)

References for published results: *Grosbois et al. 2006; †Doutrelant et al. 2000a; ‡Doutrelant et al. 2001.

### Life history traits

**Figure 1:**
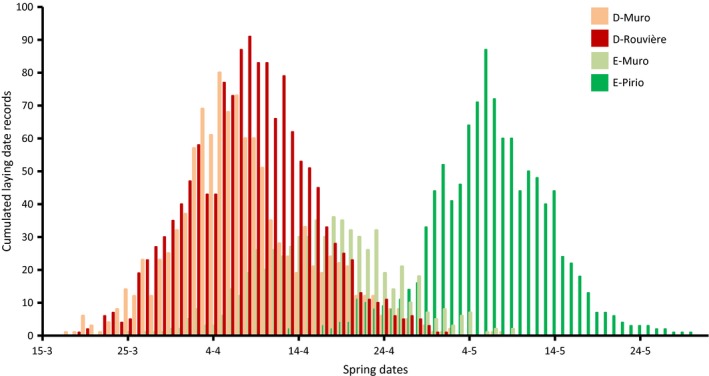
Differentiation in laying dates illustrated through the cumulated observations between 1998 and 2014 in four Mediterranean blue tit study sites.

Differences in breeding phenology are particularly striking (Lambrechts et al. 1997a; Fig. 1). As leaf‐eating caterpillars, the main prey collected for blue tit chicks, are available 1 month later in evergreen compared to deciduous forests (Blondel et al. 1999), the birds' breeding synchronization with local seasonal variation in food leads to divergent breeding phenologies even at a geographical scale much smaller than the dispersal range of the birds (Blondel et al. 1993): indeed, average laying dates are 1 month apart between D‐Muro and E‐Pirio, and 10 days between D‐Muro and E‐Muro (Table 1, Fig. 1). In fact, the breeding dates in E‐Pirio are the latest described in the whole species distribution (see the striking fig. 1 in Visser et al. 2003), which was originally quite surprising considering this population is at the southern range of the species distribution and is under a much warmer climate than most other populations included in this comparison. Interrogations on the plastic versus genetic origin of such drastic differences in the onset of laying emerged in the mid‐1980s (Blondel 1985) and led to common garden experiments in aviaries, including hand‐reared chicks (Blondel et al. 1990). These experiments suggested that differences observed in the average onset of laying had a strong genetic basis when comparing birds from mainland deciduous and Corsican evergreen habitats (Blondel et al. 1990; Lambrechts and Dias 1993). Similar attempts to test for a genetically based difference in laying date between D‐Muro and E‐Pirio were less successful, presumably because D‐Muro birds seemed to cope less well with novel and artificial environments than birds from other populations (Lambrechts et al. 1999). Concurrently to the evidence for a genetically based microgeographic variation in laying date, at least between the mainland and Corsican blue tits, plasticity was also revealed important. In these populations, as in many other bird species (Charmantier and Gienapp 2014), the timing of breeding is a plastic trait responding to various environmental cues. Early aviary tests confirmed that photoperiod is a key determining factor of timing of breeding (Lambrechts et al. 1997b; Lambrechts and Perret 2000). Further experiments revealed the role of other factors such as aviary characteristics and social environment (Caro et al. 2007), and long‐term series analyses revealed a relatively strong plastic response to spring temperature in all populations (Porlier et al. 2012a). In the context of elucidating proximate factors involved in the adjustment of the onset of breeding, our Mediterranean blue tit populations offered a valuable opportunity to test simultaneously the roles of food availability/temperature on the one hand (as historically favoured by ecologists, e.g. Perrins 1991) and photoperiod on the other (as favoured by physiologists, e.g. Silverin et al. 1993), respectively. They still offer promising perspectives for more refined aviary experiments (S. Caro, work in progress). Phenotypic differences in clutch size among the study sites are equally strong, with the E‐Pirio population presenting the lowest average clutch size recorded for this species (Table 1, Blondel et al. 1993, 1998). This variation in the number of eggs laid is well related to the abundance of food supply (Lambrechts et al. 1997a; Blondel et al. 2006), that is caterpillars, during the breeding season, yet artificial food conditions in the aviaries make it difficult to test for a genetic mechanism, as has been done for laying date (Blondel et al. 1990). Overall, classic selection analyses showed that habitat‐specific differences in the timing and abundance of food result in divergent selection for both laying date and clutch size (see detailed selection estimates in Porlier et al. 2012a) and thus confirm the possible role of habitat in driving phenotypic differences.

### Morphological traits

Blue tits from the mainland belong to the nominal *Cyanistes caeruleus caeruleus,* and are 15% larger than blue tits from Corsica, which belong to the subspecies *C. c. ogliastrae* (Table 1, Dias and Blondel 1996). Additionally, within Corsica, birds are smaller and lighter (yet with longer bills) in the poorer evergreen habitat compared to the richer deciduous habitat, with once again birds from E‐Pirio displaying extreme phenotypic values (Table 1). This habitat‐linked morphological divergence has repeatedly been shown significant for adult birds (e.g. Blondel et al. 1999) as well as for nestlings (Charmantier et al. 2004b). Interestingly, there is strong divergent selection on adult size between habitats because small adult males have a higher breeding success compared to large males in the poor evergreen forest while the reverse is true in the deciduous environment (Blondel et al. 2002). This is in contrast with results of similar selection analyses for chick morphology, which show consistent selection for larger and heavier nestlings, although with differing selection force across the study populations (Charmantier et al. 2004b). Contrarily to what has been described above for laying date, the difference in body mass (independently of adult size) measured between evergreen and deciduous habitats vanished in common garden aviary experiments (Braillet et al. 2002) as well as in cross‐fostering studies (Simon et al. 2005), suggesting a major role of phenotypic plasticity. Both caterpillar abundance and parasite prevalence were identified as key factors driving the phenotypic plasticity in morphometrics and explaining the habitat‐specific divergence with smaller and lighter birds in the poorer habitat (Blondel et al. 2006).

### Song

Blue tit song also shows marked divergence between populations (Table 1, Doutrelant et al. 2000a,b; Doutrelant and Lambrechts 2001; Doutrelant et al. 2001). The average repertoire size (number of song types produced by one male in one morning) varies among Corsican sites and is larger in these sites than in the D‐Rouviere mainland population (Doutrelant et al. 2000a). In addition, across northern Europe, blue tit song is characterized by the presence of a rapid trill (a fast series of identical notes repeated at the end of song, Bijnens 1988; Doutrelant and Lambrechts 2001; Doutrelant et al. 2001). Our mainland population fits this description as about 70% of the recorded songs contain a trill. By contrast, in Corsica, the percentage of songs possessing a trill is lower, varying between 0% (E‐Pirio) and 30% (D‐Muro, Doutrelant and Lambrechts 2001; Doutrelant et al. 2001). Overall, local variation is much more pronounced in Corsica than on the mainland, revealing the existence of different song dialects (Doutrelant et al. 2001). In particular, blue tit songs in D‐Muro and E‐Pirio display significantly different frequency range, maximum frequency, number of phrases and total duration (Table 1, Doutrelant et al. 2001), and they share very few song types (around half the repertoire if we consider the whole range of song types produced but none in common if we only consider the song types sung by more than 10 individuals, Doutrelant et al. 2001). Founder effects, isolation or drift could drive a large part of the geographic variation observed because song is a cultural trait that needs to be learned to be correctly emitted and thus is very sensitive to stochastic factors (Catchpole and Slater 2008). However, playback experiments and correlative data suggest that adaptation to both local vegetation and local interspecific competition with great tits is also a good candidate to explain the described song differences (Doutrelant et al. 1999, 2000a; Doutrelant and Lambrechts 2001).

### Personality

Very recently, the study of variation in personality using openfield protocols (Mutzel et al. 2013) and handling aggression (Class et al. 2014) scores (0–3) has revealed that these behaviours are repeatable and show strong differentiation among evergreen versus deciduous habitat patches in Corsica (G. Dubuc‐Messier, A. Charmantier & D. Réale, in preparation). In short, individuals from the deciduous island habitat (D‐Muro) show higher speed in the openfield and higher handling aggression compared to individuals from the evergreen island habitats, with similar patterns in males and females (Table 1). At the same time, we did not find any personality difference between the two evergreen island habitats for both traits (openfield exploration speed and handling aggression). These results are consistent with the pace‐of‐life hypothesis proposed by Réale et al. (2010): individuals from the rich deciduous habitat (D‐Muro) show life history characteristic of a fast pace‐of‐life (e.g. low adult survival, high reproductive investment, see Table 1) and display a personality phenotype classically associated with this kind of pace‐of‐life, that is fast exploration patterns in the openfield and higher handling aggression. The opposite is true for individuals from the evergreen habitats (E‐Muro and E‐Pirio), which are showing life history and personality phenotypes typical of a slow pace‐of‐life, demographically illustrated by lower fecundity (clutch size, number of fledglings) and higher survival rates. Similarly to the divergence observed in song characteristics, such behavioural differences across habitats could arise from restricted gene flow, and in turn could reinforce the isolation process (see more discussion on this in the mechanisms section below).

### Colour ornaments

**Figure 2:**
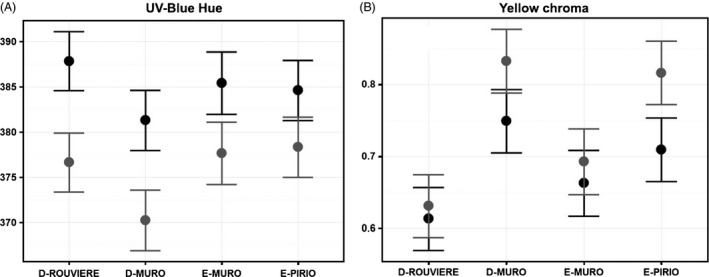
Differentiation in (A) UV/blue spectral hue of the crown patch and (B) purity (or chroma) of the yellow chest, for males (in grey) and females (in black) across our blue tit populations. Data were collected on breeding birds between 2005 and 2013. Within Corsican populations, D‐Muro birds display lower UV–blue hue compared to E‐Muro and E‐Pirio birds (values for males: D‐Muro: 370 nm, 95% CI **= [367;374]; E‐Muro: 377 nm, 95% CI **= [374;381]; E‐Pirio: 378 nm, 95% CI **= [375;382]), whereas E‐Muro birds display lower yellow chroma compared to D‐Muro and E‐Pirio birds (values for males: E‐Muro: 0.69, 95% CI **= [0.65;0.74]; D‐Muro: 0.83, 95% CI **= [0.79;0.88]; E‐Pirio: 0.82, 95% CI **= [0.77;0.86]). Means and confidence intervals are derived from linear mixed models (REML process) with age, sex and population as fixed effects and bird identity and year as random effects.

Table 1 illustrates in detail how each population of blue tits presented in Box 2 displays a unique colour phenotype (for detailed statistical results see A. Fargevieille, A. Grégoire & C. Doutrelant, in preparation) when combining measures on the blue crown patch and the yellow chest. Compared to Corsican blue tits, birds from the mainland have brighter yellow and UV–blue colorations and less dichromatic yellow chroma. Corsican populations also differ from one another. First, the chromatic values of the UV–blue coloration separate D‐Muro from E‐Muro and E‐Pirio: deciduous birds display more UV (lower hue and higher UV chroma) and are more dichromatic (Fig. 2). Second, the chromatic values of the yellow coloration separate E‐Muro from D‐Muro and E‐Pirio: the yellow chroma of E‐Muro birds is lower and less dichromatic. Finally, the population of E‐Pirio shows different brightness patterns, with duller UV–blue coloration and brighter yellow coloration. More studies are needed to understand the origin of these population differences and whether or not they are adaptive, but it is noteworthy that D‐Muro and E‐Muro birds differ so strongly in both UV–blue and yellow coloration. These differences may contribute to the observed genetic differentiation if mate choice preference for colour signal differs between these populations or if individuals do not settle randomly in respect to their coloration. In the face of a habitat‐specific divergence at a micro‐geographic scale, the observed differences in the UV–blue coloration between the two Muro populations are particularly interesting to illustrate this possible divergent intrasexual selection. Indeed, the blue crown patch is involved in social and intrasexual interactions (Rémy et al. 2010; Midamegbe et al. 2011), and as the deciduous forest of D‐Muro offers a richer breeding habitat than the evergreen forests of E‐Muro and E‐Pirio, birds in D‐Muro might display more UV because they are socially dominant over the birds breeding in evergreen patches (Braillet et al. 2002). Another hypothesis, involving plasticity in coloration, is that the stronger UV in D‐Muro results from a lower cost of breeding in this population. We indeed experimentally demonstrated that increasing the cost of reproduction affects the parents coloration in the following year (Doutrelant et al. 2012).

## The phenotypically differentiated traits are heritable

In the context of testing whether the strong phenotypic differentiation described in the previous section can be due to microevolution leading to local adaptation, an important albeit brief point we make here is that nearly all morphological and reproductive traits in Table 1 have been shown to be heritable, either in our populations, or in other blue tit studies. Based on an extensive review of heritability estimates published between 1974 and 2011 (see online supplementary material of chapter 2 in Postma 2014), average heritability in blue tit studies was 0.380 (SE = 0.353, *n *= 46 estimates) for morphological traits, 0.261 (SE = 0.221, *n *= 7) for life history traits, −0.005 (SE = 0.078 *n *= 2, dispersal distance) for behavioural traits and 0.669 (SE = 0.505, *n *= 7) for physiological traits. When focusing on published estimates derived from the Mediterranean populations described in Box 2, we found evidence for significant heritability in laying date (range of heritability: 0.20–0.43, Caro et al. 2009), body mass (0.267–0.638, Charmantier et al. 2004b; Teplitsky et al. 2014b), tarsus length (0.418–0.597, Charmantier et al. 2004b,c; Teplitsky et al. 2014b), wing length (0.216–0.319, Teplitsky et al. 2014b) and nonsignificant heritability in adult survival probability (heritability = 0.018, CI = [0.000; 0.077], Papaïx et al. 2010). As a note of caution, these heritability estimations can vary quite substantially depending on the environmental conditions (Charmantier and Garant 2005), as well as on the complexity of the models being fitted (Wilson 2008). Also, they were all based on animal models run on a social pedigree; hence, they might be underestimated because of frequent extra‐pair paternities in our populations (Charmantier et al. 2004a; Charmantier and Réale 2005). Apart from survival, all other morphological and life history traits in Table 1 are classically shown to be heritable in avian species (Postma 2014), which does not preclude strong plasticity in many of these traits, as has been discussed above (see the roles of photoperiod and timing of food abundance for breeding phenology and clutch size). Although quantitative genetic estimates on behaviour and colour ornament traits are much more recent and still scarce, there is now good evidence for strong additive genetic variance in blue tit and great tit personality traits such as handling aggression and exploratory behaviour (e.g. Dingemanse et al. 2002; Drent et al. 2003; Class et al. 2014), while much more moderate heritability for blue tit colour ornaments (Johnsen et al. 2003; Hadfield et al. 2006; Drobniak et al. 2013). To date, the cultural and genetic heritability of blue tit song has not been quantified and this remains an important step for future research in order to understand the observed differences (Wilkins et al. 2013).

## Mechanisms involved in the onset and maintenance of phenotypic divergence

Adaptive divergence across populations has long been considered conditional on a) antagonistic selection across habitats and b) a physical isolation between divergent populations, thereby restricting gene flow (Wright 1943; Maynard Smith 1966). Both theoretical (e.g. Garcia‐Ramos and Kirkpatrick 1997) and empirical evidence (e.g. Hendry et al. 2002) supported this view for almost half a century. Our overall understanding of adaptive divergence today includes much more complexity. For instance, it is now recognized i) that restricted gene flow can either be a cause or a consequence of adaptive diversification (Räsänen and Hendry 2008), ii) that in some instances, gene flow can induce rather than constrain phenotypic divergence (Guillaume 2011) and iii) that local adaptation can occur at a very fine spatial scale despite gene flow (see e.g. Muir et al. 2014; Langin et al. 2015; Moody et al. 2015). In a recent review, Richardson et al. (2014) discuss a set of mechanisms that can initiate divergence or reinforce it even in the presence of gene flow. Here we review evidence in our system for three of these nonexclusive mechanisms: habitat selection, selective barriers against migrants and positive assortative mating.

### Habitat selection

There are several lines of evidence suggesting that a process of matching habitat choice (Edelaar et al. 2008) could cause directed gene flow between forest habitat types. The fact that birds from E‐Pirio are smaller and lighter than birds from D‐Muro allowed Blondel and colleagues (fig. 2 in Blondel et al. 1999) to provide the first evidence for nonrandom gene flow as immigrants showed more similar morphometrics compared to resident birds than expected by chance, suggesting that birds born in deciduous habitats would prefer to breed in deciduous habitats, and *vice versa* for birds from evergreen habitats. Later on, a study of feather isotopic signatures in the main study sites presented in Box 2 partly confirmed such habitat choice, while also revealing in each area a set of immigrant birds with outlier isotopic signatures, presumably originating from oak void habitats (Charmantier et al. 2014). Further investigations are underway to refine the definition of isoscapes within and around our study populations, in the aim of identifying the landscape origin of each immigrant bird (C. de Franceschi, work in progress). This would allow testing for a matching habitat choice process, for example to test the prediction that blue tits with fast exploratory behaviour preferentially disperse to deciduous forest patches while ‘slow’ birds prefer to breed in evergreen patches.

### Selective barriers against migrants

Richardson et al. (2014) describe this mechanism as reduced fitness for migrants, acting before they reproduce through premating isolation. The habitat‐specific breeding phenology could well work as a reinforcing barrier against maladapted dispersal if laying date is culturally or genetically inherited. Indeed, if two populations are breeding at different spring periods, they will obviously not mate with each other, which in turn will strengthen the reproductive barrier that may have arisen for other independent reasons. Anecdotic cases of birds born in deciduous habitats but breeding in evergreen ones support this view, as these birds initiated reproduction too early compared to the caterpillar abundance (Charmantier, pers. comm.), and vice versa for the reverse situation. Apart from this phenological mismatch between habitat types, it has also been suggested that differences in social dominance of birds originating from deciduous or evergreen habitats could contribute to restricting dispersal and maintaining population phenotypic differentiation at a micro‐geographic scale (Braillet et al. 2002). Indeed, the larger size of male blue tits from D‐Muro (and possibly stronger colorations) provides them with a systematic dominance over the smaller males of E‐Pirio, thereby creating a potential barrier for the settlement of evergreen birds in deciduous areas. Finally, the dialects and differences in avian songs detailed previously could also contribute in driving population divergence, such as what has been shown in species where females prefer to mate with the males possessing the local versus a foreign dialect (e.g. Podos 2010). Hence, the existence of dialects in Corsica could contribute to explain the reduced gene flow between valleys, previously highlighted as part of an insular syndrome (Blondel et al. 1999). In the future, we aim at testing this crucial hypothesis using both mate choice experiments and playbacks.

### Positive assortative mating

**Figure 3:**
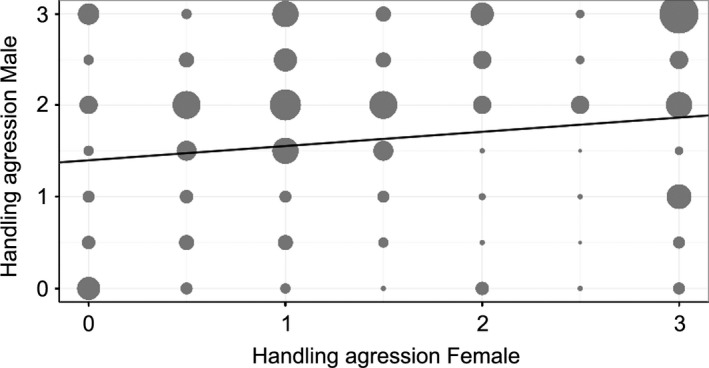
Relationship between handling aggression scores (0 to 3, with 0 no aggressive behaviour and 3 maximum aggressiveness) of male and female social partners in Corsican populations (E‐Pirio, D‐Muro and E‐Muro) from 2011 to 2014; the slope (0.156, 95% CI **= [0.054;0.260]) and intercept (1.400, 95% CI = [1.084;1.751]) of the line are derived from a linear mixed model (Bayesian framework) with females handling aggression score as response variable, partner handling aggression score and year as fixed effects and female identity, partner identity and handling aggression observer as random effects; *n* = 336 females, 345 males, 10 observers. The size of the points refers to the number of pairs with a given combination of handling aggression scores (min = 2, max* *= 24).

After they settle in a new habitat, maladapted immigrants can still have a restricted contribution to the local gene pool because of sexual selection processes (Richardson et al. 2014). In particular, positive assortative mating may discriminate against rare phenotypes. In Corsica, we found support for a positive assortative mating in handling aggression scores (estimated correlation between male and female handling aggression: 0.156, 95% CI = [0.054, 0.260]; Fig. 3) and for exploratory speed in the openfield for young females of 1 and 2 years (estimated correlation: 0.280, 95% CI = [0.073, 0.473], *n *= 41 females, 38 males; 2011–2014). Similar assortative mating for personality has been identified in several bird species, including great tits (Groothuis and Carere 2005) and zebra finches *Taeniopygia guttata* (Schuett et al. 2011). Assortative mating for UV–blue and yellow coloration is also around 30%, while varying across years (A. Fargevieille unpublished data). Such assortative mating also suggests a potential role of sexual selection in the observed phenotypic differentiation, which needs further exploration in future work.

## Isolation by environment revealed by population genetics and genomics

Throughout the duration of the project, it was established that an impressive number of phenotypic traits diverged between the mainland and the island of Corsica, but also at small spatial scales within Corsica (Table 1). Moreover, for a large number of these often uncorrelated traits, vegetation type was systematically identified as the driver for phenotypic differentiation. Given that significant additive genetic variance was reported for several of these traits, it is pertinent to ask whether there is a habitat‐driven genetic basis for the systematic differences observed at the phenotypic level. As discussed above, aviary and cross‐fostering experiments provided evidence for a genetic basis in the habitat‐specific differentiation of some of the traits (in particular laying date), but not others (e.g. morphology, clutch size), even though all traits were heritable. In this study system, testing whether blue tit genetic differences are driven by habitat type [defined as isolation by environment (IBE, Wang and Bradburd 2014), isolation by ecology (Edelaar and Bolnick 2012) or more broadly genotype–environment associations (GEA, Hedrick et al. 1976)] therefore becomes a necessary stepping stone before identifying the genetic basis of local adaptation (Barrett and Hoekstra 2011).

Gaining an in‐depth knowledge of population genetic structuring has recently witnessed immense progress thanks to next‐generation sequencing (NGS) methods (Davey et al. 2011). For our study system, we could thus transition from a panel of 6–10 highly polymorphic, neutral microsatellite markers (Porlier et al. 2012b) to up to *c*. 12 000 bi‐allelic single nucleotide polymorphism (SNP) markers (Szulkin *et al*, in press). SNP markers were generated through a RAD seq (restriction site associated DNA sequencing) approach (Baird et al. 2008) and positioned in both coding and noncoding regions of the genome. Thus, 197 resident birds from D‐Rouviere and the three Corsican populations (D‐Muro, E‐Muro and E‐Pirio) were genotyped using single‐end RAD sequencing (Szulkin *et al*, In press). The distribution of allelic frequencies revealed by the SNP marker data set differed between the mainland and Corsica and was characterized by a larger number of rare allelic variants in Corsica when compared to the mainland – a variability that will need to be addressed in future population genomic work. Using a series of complementary population genetics and landscape genetics analyses, both microsatellite and SNP‐based studies found multiple evidence of habitat‐linked genetic differentiation (Porlier et al. 2012b; Szulkin *et al*, in press, see Table 2), which we refer to as isolation – by – environment (IBE, Wang and Bradburd 2014). Importantly, SNP‐based analyses also found a small yet highly significant genetic differentiation, measured with pairwise *F*st tests, between D‐Muro and E‐Muro (Table 2). This result was further corroborated by several complementary population genomic analyses. Given that the two study sites are only 5.6 km apart and that blue tit average dispersal distance is believed to range between 330 m and 4 km (depending on dispersal distance estimation method (Tufto et al. 2005; Ortego et al. 2011) while maximum dispersal values are known to range up to 470 km (Winkel and Frantzen 1991), the genetic distinctiveness of the two populations is remarkable. Overall, an important question that thus needs to be further addressed is whether genetic IBE results from reduced dispersal through habitat choice, local selection against maladapted genotypes or a combination of both. Research in progress will focus on combining phenotypic and genetic data to improve our understanding of the ecological and evolutionary processes shaping observed patterns of genetic differentiation.

**Table 2:** Above the diagonal: *F*st values from Porlier et al. (2012b, data averaged across several years), *n *= 247 Individuals, 6–10 microsatellite markers. Below the diagonal: *F*st values for SNPs retained after filtering with 5% MAF and a 95% call rate. *n *= 197 individuals, 3159 SNPs (Szulkin *et al*, in press). Empirical *P*‐values in brackets were computed using 500 permutations (lowest *P*‐values are therefore bounded by 0.002)

	D‐Rouvière	D‐Muro	E‐Muro	E‐Pirio
D‐Rouvière	–	0.049	0.042	0.041
D‐Muro	0.0541 (*P *≤ 0.002**)	–	0.007	0.004
E‐Muro	0.0335 (*P *≤ 0.002**)	0.0156 (*P *= 0.004**)	–	0.005
E‐Pirio	0.0520 (*P *≤ 0.002**)	0.0099 (*P *= 0.347)	0.0102 (*P *= 0.403)	–

The possibility of combining pedigree‐based quantitative genetics with genomic data is indisputably an exciting new analytical framework to study heritability (Stanton‐Geddes et al. 2013; Berenos et al. 2014) and variation of phenotypic traits in our blue tit study system. Assuming a sufficiently dense marker density, the complementary use of SNP‐based relatedness estimators and field‐collected pedigrees should allow to accurately perform not only quantitative genetic analyses, but also to identify regions of the genome that have undergone selection (Jensen et al. 2014). Several methods have been developed to meet these goals: for example, QTL‐focused approaches (recently identified as prone to type one error in the context of field‐based studies with limited sample size, Slate 2013) are being replaced – or complemented by – new methods for phenotype‐associated genomic prediction (Goddard and Hayes 2009), genomic partitioning (Robinson et al. 2013; Santure et al. 2013), as well as relatedness‐ and population structure‐corrected genomewide association studies (GWAS) and candidate gene‐based approaches (Savolainen et al. 2013).

With the decreasing costs of sequencing and the development of sequencing platforms capable of generating even longer sequence reads at lower cost, SNP data sets are likely to be replaced with sequence (haplotype) data and even whole genome sequencing in the future. While genotype‐by‐sequencing approaches will allow us to substantially improve the availability of reference genomes, alignment and ascertainment bias‐free allelic frequency data, some challenges will also lie ahead: bioinformatics capacity in terms of processing power and know‐how needs to be regularly updated. In addition, analytical lines of research will always have to be balanced with time spent in the field – a cornerstone stage necessary for the collection of phenotypic, fitness and environmental data relevant to our study species of interest.

## Conclusions, limitations and perspectives on adaptive divergence

We have reviewed here evidence strongly suggesting that the striking phenotypic differentiation in ecologically relevant traits exhibited by natural populations of blue tits in their southern range‐edge putatively reflects adaptation to the heterogeneity of their habitat. This is not exclusive to a concomitant role of plasticity (as has been discussed for laying date and body mass), bearing in mind that plasticity itself can vary across populations (Porlier et al. 2012a), can be adaptive and can evolve. The conclusions presented here are the result of 40 years of individual monitoring in contrasted habitats and of collection of data on a diverse set of phenotypic traits, including estimations of fitness, on individual relatedness and on genetic and genomic variation. This long‐term effort has provided compelling evidence for a habitat‐driven phenotypic variation that is most likely driven by local adaptation: laying date is a heritable trait, which shows contrasted distributions and divergent selection across evergreen and deciduous habitats, and these differences are partly maintained in aviary conditions. However, we acknowledge here that these clear conclusions come with certain limitations which will inspire our future research. First, our study system is dependent on the natural heterogeneity of the habitat, and as such, it has some inherent limitations. In particular, there is a lack of replication of deciduous landscapes in Corsica because of its rarefaction in the Mediterranean region. Second, studying a variety of traits in natural bird populations is not easily combined with common garden and/or reciprocal transplant experiments, which makes it more difficult to provide evidence that observed phenotypic differences have a genetic basis. Third, divergent selection has not yet been illustrated (nor explored) for all the traits presented in Table 1. Fourth, some of the phenotypic variation observed between our study sites (e.g. differences in yellow chroma across Corsican populations) is not clearly linked to habitat or geographic distance. In these rare cases where no ecological driver was yet identified, the most parsimonious explanation for this phenotypic variation remains that it is explained by nonadaptive processes such as drift (even if, in the specific case of the yellow chroma, the literature on the evolution of colours suggests that a finer exploration of factors such as parasites, food abundance or environmental stressors will probably reveal that some of these factors may explain the observed population differences). Two of the most relevant and promising perspectives to overcome these limitations are the multidimensional study of (natural, social and sexual) selection across habitats and the development of an ecological genomics approach.

In a recently developed theoretical model, the strength of local adaptation is shown to be correlated to trait dimensionality (Macpherson et al. 2015): local adaptation (or the fitness advantage of residents over immigrant individuals) increases with the number of traits under spatially variable selection, in other words the number of traits influencing adaptation to environmental heterogeneity. Although we have presented here a phenotypic divergence measured on many different traits, some of these traits, in particular the timing of breeding, display stronger differences across populations than others. A comparative analysis across traits could offer a comprehensive view on which traits are more strongly habitat related. Also, as many of these traits are correlated, further work on the evolution of habitat‐specific trait combinations should integrate estimations of phenotypic and genetic covariances (Teplitsky et al. 2014a). Finally, the recent development of new approaches for the study of the dynamics of selection in space and time and its impact on adaptive evolution (Bell 2010; Siepielski et al. 2013; Chevin and Haller 2014) makes the estimation of spatial variation in selection for all traits in Table 1 a timely and exciting perspective which would greatly contribute to understanding differences in adaptive divergence rates.

It has long been acknowledged that loci involved in adaptation should show increased levels of genetic differentiation between populations (Lewontin and Krakauer 1973; Nielsen 2005). Benefiting from that idea, several methods have been proposed to detect loci under selection (e.g. Nielsen 2005; Excoffier et al. 2009; Narum and Hess 2011). Our next step in this project is to explore further the genomic differentiation across temporal, spatial or ecological boundaries and attempt to pinpoint genomic regions that have been involved in adaptation. While it will be impossible to identify all loci responsible for phenotypic variation (Manolio et al. 2009), new tools are being developed to most accurately capture loci with larger effects. Based on the strong phenotypic divergence described in this article, we are particularly excited at the perspective of examining whether outlier loci co‐localize with candidate genes that have been described for the timing of breeding (Johnsen et al. 2007; Caprioli et al. 2012; Liedvogel et al. 2012) and for personality (Savitz and Ramesar 2004; Fidler et al. 2007; Mueller et al. 2014). Such data will also allow to answer the crucial question of whether these focal traits are affected by the same molecular mechanisms in different populations and different ecological contexts.
